# International Spinal Cord Injury Fracture History Extended Data Set

**DOI:** 10.46292/sci25-00025

**Published:** 2025-08-22

**Authors:** Leslie R. Morse, William A. Bauman, B. Catharine Craven, William D. Leslie, Thomas J. Schnitzer, Karen Troy, Fin Biering-Sorensen

**Affiliations:** 1 Department of Physical Medicine and Rehabilitation, University of Miami Miller School of Medicine, Miami, Florida; 2 James J. Peters VA Medical Center, National Center for the Medical Consequences of SCI, Bronx, New York; 3 Neural Engineering and Therapeutics Team, KITE Research Institute - University Health Network, Department of Medicine, University of Toronto, Toronto, Ontario, Canada; 4 Department of Internal Medicine, University of Manitoba, Winnipeg, Manitoba, Canada; 5 Department of Physical Medicine and Rehabilitation, Northwestern University Feinberg School of Medicine, Chicago, Illinois; 6 Department of Biomedical Engineering, Worcester Polytechnic Institute, Worcester, Massachusetts; 7 Department of Clinical Medicine, University of Copenhagen, Copenhagen, Denmark; 8 Department for Brain and Spinal Cord Injuries, Bodil Eskesen Center, Rigshospitalet, Copenhagen, Denmark

**Keywords:** fracture, osteoporosis, quantitative computed tomography, spinal cord injury

## Abstract

**Objectives:**

The objective of the study is to develop the International Spinal Cord Injury (SCI) Fracture History Extended Data Set within the framework of the International SCI Data Sets to permit consistent collection and reporting of fracture history in the SCI population.

**Methods:**

The International SCI Fracture History Extended Data Set has been developed by a working group. The initial data set was open for 2 months for discussion and was revised based on suggestions from members of the International SCI Data Sets Committee, the International Spinal Cord Society (ISCoS) Executive and Scientific Committees, American Spinal Injury Association (ASIA) Board, other interested organizations, societies, and individual reviewers. The data set was also posted for 2 months for comments on ISCoS's and ASIA's websites.

**Results:**

The final data set contains questions on fractures after SCI. Because the information may be collected at any time, the date of data collection is important to capture relative to the time lapsed after SCI. The data set includes information on fracture history (location, etiology, treatment, complications for each fracture event), osteoporosis treatment (current and past use), bone measures by quantitative computed tomography (6 variables), and body composition (7 variables). The complete instructions for data collection and the data sheet itself are freely available on the ISCoS website (https://cdn.ymaws.com/www.iscos.org.uk/resource/resmgr/fracture/iscieds_fracture_1.pdf).

**Conclusion:**

The data set proposes to collect information on bone loss, other factors potentially predictive of fracture risk, and fracture in persons with SCI to guide clinical management and future research activities.

## Introduction

The International Spinal Cord Injury (SCI) Fracture Extended Data Set was created to standardize the collection and reporting of key clinical and demographic factors and diagnostic imaging outcomes related to osteoporotic fractures according to the best practices and processes established by the International SCI Data Sets.^1^ This data set was produced under the auspices of International Spinal Cord Society (ISCoS) and the American Spinal Injury Association (ASIA) and in cooperation with the International Society for Clinical Densitometry (ISCD). Any major changes will be brought back to each respective executive committee for review and approval. This data set is appropriate for use among patients with traumatic or nontraumatic etiologies of upper motor neuron lesions of the spinal cord as well as conus medullaris and cauda equina lesions.

Osteoporotic fracture is common after SCI and is associated with loss of function and subsequent medical complications including pressure injury, infection, fracture nonunion or malunion, and amputation. Fractures are associated with increased 5-year mortality^2^ and may limit the ability to utilize neurorestorative technologies, including robotic exoskeletal-assisted walking. There have been recent advances in the clinical approach to sublesional osteoporosis in SCI with the publication of the following 3 complementary consensus-based documents: (1) Bone Mineral Density Testing in Spinal Cord Injury: 2019 ISCD Official Position,^3^ (2) Bone Health and Osteoporosis Management in Individuals with Spinal Cord Injury Clinical Practice Guideline,^4^ and (3) Acute Lower Extremity Fracture Management in Chronic Spinal Cord Injury 2022 Delphi Consensus Recommendations.^5^ These guidelines provide SCI-specific evidence-based clinical guidance for dual-energy absorptiometry (DXA)–based diagnosis and monitoring of osteoporosis,^3^ therapeutic options for prevention and treatment of osteoporosis,^4^ and fracture detection and management.^5^ Despite these advances, osteoporotic fractures continue to be a common complication, suggesting the need for widespread implementation of a standardized clinical approach to osteoporosis and for additional clinical research. Standardization of clinical and research outcomes, multicenter collaborations, and large, well-designed clinical trials or comparative efficacy trials that test novel antiresorptive or osteoanabolic therapeutics are greatly needed.

Several SCI-specific non-BMD risk factors for osteoporosis have been proposed, but more research is needed to refine and improve fracture risk prediction based on diagnostic imaging (DXA or quantitative computed tomography [QCT]), metabolic bone markers, and clinical/demographic factors. The proposed data to be collected in this data set are expected to advance fracture risk prediction algorithms in persons with SCI, standardize clinical trial bone outcomes, and provide an evidence-based approach to rehabilitation strategies to reduce fracture incidence. Therefore, this data set is intended for use among clinicians and researchers for assessment of prevalent and incident fractures and factors (ambulatory status, medication use, putative osteogenic therapies, health habits, and medical comorbidities) that may be associated with an increased risk of fracture. This extended data set expands upon factors assessed in the International SCI Endocrine and Metabolic Extended Data Set and includes additional imaging variables (measurements of the lower extremities by QCT and regional body composition measures of the skeleton and soft tissue by DXA) for standardization across research protocols.

The International SCI Fracture Extended Data Set was developed to complement and be used in conjunction with the International SCI Core Data Set^6^ that provides demographic and injury information, the Spinal Cord Independent Measure (SCIM) mobility tool to assess mobility,^7^–^9^ the International SCI Endocrine and Metabolic Extended Data Set that includes calcium metabolism and DXA outcomes,^10^ and the following International SCI Basic Data Sets that detail medical comorbidities: endocrine and metabolic,^11^,^12^ cardiovascular function,^13^ pulmonary function,^14^ and musculoskeletal.^15^ While the musculoskeletal dataset includes information on fragility fracture, the SCI Fracture Extended Dataset provides additional detailed information to guide clinicians and inform research.

## Materials and Methods

The members of the International SCI Fracture History Extended Data Set working group have extensive experience in both the clinical management and the clinical research of endocrine and metabolic challenges associated with SCI. The validity of the selected measures is based on the SCI literature and expert opinion. To ensure consistency in the data collection and facilitate interpretation, a detailed operative definition and related use information is provided in a syllabus for each specific variable.

The data sheet is available on the ISCoS website (https://cdn.ymaws.com/www.iscos.org.uk/resource/resmgr/fracture/iscieds_fracture_1.pdf). Listed below are the variables included in the fracture history extended data set.

### Date of data collection

Date of data collection is required to calculate injury onset and to provide chronological information regarding data elements collected for the same individual at other time points and when comparing information collected to use in other data sets.

### Fracture history

Fracture history will assess the skeletal site, mechanism of injury, medical management, and known complication(s) for each fracture. Limited information exists on factors associated with incident fracture risk and the prevalence of fracture-related complications after SCI.^16^ Potential complications include delayed union, nonunion, pressure injury, deep venous thrombosis, autonomic dysreflexia, new contracture, loss of range of motion, increased spasticity, infection, and amputation. Wide fracture treatment variations are known to exist^17^ despite recently developed fracture management guidelines.^5^ Therefore, recording fracture treatment details is recommended (surgery, bracing, casting, bedrest, medication, or no treatment). According to the 2019 ISCD SCI-specific position statements,^3^ osteoporosis diagnosis should be made in an individual with SCI after any low-impact fracture following a work-up to exclude other secondary causes of osteoporosis. Based on the mechanism of injury, fracture etiology will be classified into one of the following three categories:

1. *Fragility fracture*: Fractures that occur without an identifiable trauma or event due to turning over in bed, a caught foot wheeling through a doorway, a dropped object on the body; during stretching/physical therapy, a fall from a wheelchair, a fall from standing height or less; during weight bearing or assisted ambulation activities (exoskeletal-assisted walking, manual or robotic bodyweight supported treadmill training, overhead harness systems, functional electrical stimulation [FES], epidural spinal stimulation), or due to another cause to be specified.
2. *Traumatic fracture*: Fractures due to a fall from greater than standing height, sports injury, motor vehicle/motorcycle accident, or other cause to be specified.
3. *Unable to determine* etiology.

### Fracture Risk Assessment Tool

The Fracture Risk Assessment Tool (FRAX) will estimate 10-year fracture risk based on clinical risk factors and (optionally) bone density at the femoral neck.^18^ Fracture rates vary widely among countries, and this has led to the creation of country-specific FRAX calculators. For some countries, there are several race-/ethnicity-specific calculators. Therefore, input variables and the specific FRAX calculator used will be recorded to compare and interpret results across regions. The FRAX calculator can be found at: http://www.shef.ac.uk/FRAX/. Country-specific calculator tools can be found by following the link and choosing the tab “Calculator Tool” and the country of interest. If the country of interest is not represented, the country that most closely resembles the epidemiology of the population with osteoporosis in the country of interest should be chosen from the list.

The FRAX algorithm has not been validated in the SCI population. The degree to which completeness of neurological impairment and associated degree of immobilization in those with SCI factor into the prediction of sublesional osteoporosis or fracture risk is largely unknown. Furthermore, the FRAX algorithm considers bone density at the hip; it is unknown if this tool will predict fractures at the knee (distal femoral metaphysis and proximal tibial metaphysis). A significant relationship exists over a wide range of values between the hip and knee, but the correlation appears less strong with greater loss of bone at the knee.^19^ Therefore, the prognostic utility of bone density at the knee for fracture risk prediction needs to be better studied.

Of note, there is a proposed SCI-specific fracture risk prediction algorithm^20^ that has yet to be validated. The FRAX model is calibrated for adults aged 40 to 90 years. Severe osteoporosis develops in adults with SCI under the age of 40, and it is possible to calculate a FRAX score entering an age below 40 years. However, the tool will predict the probability of fracture at age 40. While there is some evidence that FRAX performs better in men with SCI over the age of 50 and postmenopausal women with SCI,^21^ more research is needed to determine the value of the FRAX algorithm for fracture prediction in adults with SCI across the age spectrum.

### Osteoporosis treatment

This variable will assess previous (over the last 12 months) and/or current use of medications to treat osteoporosis, medications that potentially affect bone metabolism, and osteogenic physical therapies. Therapy frequency and average daily dose will also be recorded. Osteoporosis medications, including bisphosphonates,^22^–^26^ romosozumab (a soluble antibody against sclerostin),^27^ parathyroid analogues,^28^–^30^ and denosumab (a soluble antibody against receptor activator of nuclear factor kappa-B ligand [RANKL]),^31^–^34^ have been studied in SCI. Additionally, studies have shown new bone formation or reduced bone loss in response to electrical stimulation,^35^ FES biking,^36^ and FES or vibration therapy after SCI.^37^ The average daily dose and duration of use of all therapeutic interventions should be recorded.

### QCT-derived bone measures

Clinical trials and other research targeting bone health after SCI would benefit from a higher level of detail and precision than that routinely obtained in delivery of clinical care. If available, it is recommended that QCT measures, rather than or in addition to DXA measures, be adopted as a primary outcome for clinical trials that address questions related to osteoporosis treatment in persons with SCI.^38^ Bone density assessment by DXA is widely used clinically, is cost-effective, and has been shown to discriminate between those with and without fractures after SCI.^39^ However, CT-based measures have recently been suggested to outperform DXA measures in discriminating between individuals with and without a prevalent fracture history.^40^ Therefore, we recommend QCT-derived measures as an adjunct to the DXA data collected in the metabolic dataset. For each variable, the distal-most 30% of the femur or the proximal-most 30% of the tibia is suggested for analysis, but regional acquisition may vary, requiring the documentation of the precise skeletal site(s) acquired. Peripheral QCT is generally limited to imaging of the tibia because imaging the distal femur remains technically challenging. Because of the appreciated limitation in acquiring regions of interest with peripheral QCT, QCT measurement has been included in the current dataset.

#### Bone volume

This variable will assess integral (everything within the periosteal surface), cortical, and trabecular bone volume at skeletal sites of interest, including distal femur and proximal tibia. In most cases, unilateral scans are sufficient and balance cost/radiation exposure and data collection burden. Decisions to obtain bilateral knee scans may be made based on muscle/strength asymmetry.

#### Volumetric bone density

This variable will assess integral (everything within the periosteal surface), cortical, and trabecular volumetric bone density at skeletal sites of interest.

#### Volumetric bone mineral content

This variable will assess integral (everything within the periosteal surface), cortical, and trabecular volumetric bone mineral content (BMC) at skeletal sites of interest, including distal femur and proximal tibia.

#### Torsional strength index

Given that torsional (spiral) fractures are commonly observed after SCI,^41^,^42^ torsional stiffness is an accurate and clinically relevant outcome.^43^

#### Mass-weighted principal moments of inertia of the cross section

This variable will assess the resistance to bending about the axes for which the bone is both strongest and weakest at skeletal sites of interest, including distal femur and proximal tibia.

#### Cross-sectional area

This variable will assess the cross-sectional area at skeletal sites of interest, including distal femur and proximal tibia.

### Body composition by DXA

Muscle–bone interactions have been defined to a limited extent in persons with SCI. The magnitude of the loss of total body lean mass in those with SCI was correlated with the magnitude of the loss of total body or leg BMC.^44^ An association between muscle and lower extremity bone density^45^ or bone quality^46^ has been reported after SCI. Adipose tissue is a major regulator of bone metabolism.^47^,^48^ In persons with SCI, a direct association was reported between total body percent fat and leg bone mineral density (BMD), and leg fat mass was the single most significant predictor of leg BMD or leg BMC.^49^ Visceral fat is metabolically active and is a source of adipose derived hormones, including leptin and adiponectin, which can modulate bone metabolism.^45^,^50^–^61^ Android fat is considered an indicator of visceral fat.^62^ Therefore, DXA-derived body composition measures of soft tissue are recommended to be obtained as an adjunct to the DXA data collected in the metabolic dataset.

#### Lean mass

This variable will assess lean mass at skeletal regions of interest, including total body, arms, and legs.

#### Percent fat mass

This variable will assess percent fat mass at skeletal regions of interest, including total body, gynoid region, and android region. This variable will also assess visceral adipose tissue (VAT) area (cm^2^). DXA software is used to define standard gynoid and android regions. FDA-approved software is available for VAT area assessment by DXA. The lower boundary of the android region is the cut through the pelvis at the level of the iliac crests. The upper boundary of the android region extends upwards to 20% of the distance between the pelvis and the base of the skull and the lateral boundaries are the arm cuts. The upper boundary of the gynoid region below the pelvis is the cut that extends downward from 1.5 times the height of the android region. Lateral boundaries of the gynoid region are the outer leg cuts. Percent fat mass in each region is reported as the total of the percent fat in the right and left sides.

## Discussion

The International SCI Fracture Extended Data Set is intended to standardize documentation of the risk factors, complications, and treatments for osteoporotic fracture. Although QCT assessments of bone or body composition assessments by DXA may not be universally accessible clinically, the data set does give a structured, standardized, clinically relevant framework for capturing fracture etiology, management, complications, and osteoporosis treatment. Clinicians may use those elements to guide documentation. History of osteoporotic fracture is an important factor when making a clinical decision with respect to the safety and appropriateness in the prescription of neurorestorative therapies, including that of assisted ambulation.

Future clinical trials are needed with osteoporotic fracture being the primary endpoint, an experimental approach that may provide the opportunity to address more directly the predisposing factors for fracture in the SCI population. This data set is complementary to existing data sets and has both clinical and research applicability. More research is needed to validate the data set and to implement its use in clinical and research settings.
